# Marine microbiome as source of natural products

**DOI:** 10.1111/1751-7915.12882

**Published:** 2017-10-27

**Authors:** Fernando de la Calle

**Affiliations:** ^1^ Department of Microbiology R&D Pharma Mar S.A. Avda. de los Reyes, 1. Colmenar Viejo 28770 Madrid Spain

Less than 1% of living microorganisms can be cultured in the laboratory, but even this minute part has produced incredible discoveries such as antibiotics that have saved millions of lifes. We can only imagine what other great inventions will come to light when the rest of this enormous universe of living genomes is awakened. Everything is recorded in the genes, and modern metagenomic studies, involving the large‐scale sequencing of the genetic material of marine microorganisms, as well as the similarities between the human gut and ocean microbiomes, are not only providing powerful insights into why the Earth is a living planet, but are also revealing possible causes and promising cures for metabolic diseases, including cancer. The marine microbiome contains many billions of genes with the ability to express an unimaginable arsenal of small molecules, as well as proteins, lipids and others classes of products with ecological functions that can be ‘humanized’ for the discovery of new pharmaceuticals, improved enzymes, biopolymers and novel biomaterials, as well as new resources for food and feed stocks, nutraceuticals, diagnostic devices, personal care and cosmetic products and an ever‐increasing list of marine natural products. Today, the term Marine Biodiversity is known as Marine Genomic Resources.

Whilst ‘Blue Biotechnology’ remains in its infancy, a report for European DG Maritime Affairs and Fisheries (2014) (http://ec.europa.eu/dgs/maritimeaffairs_fisheries/consultations/index_en.htm) projected that revenue in Europe from blue biotechnology could reach €1 billion within 5 years if a market growth of 6–8% per annum was maintained and result in creation of 10 000 new jobs. A 2015 report analysis of market for marine biotechnology from Smithers Rapra (http://www.smithersrapra.com/news/2015/October/market-for-marine-biotechnology) indicated that this global market has the potential to reach $4.8 billion by 2020, rising to $6.4 billion by 2025. However, in the main, these estimations are based on ‘classical’ approaches such as aquaculture, bioenergy and algal cosmetics and underestimate the role of the marine microbiome. Market opportunities and job creation are likely to significantly increase in the future with the advent of transversal technologies directed at exploiting other biotechnological advances, such as the evolution of bioinformatics, synthetic biology, molecular diagnostics and devices, biocatalysis and the many OMICS technologies. The marine microbiome must be an essential part of the bioeconomy. However, whilst the enormous potential of the marine microbiome has been recognized by industry, there is currently a lack of coordination amongst policy makers, governments, civil society organizations, academia and large industries, perhaps due in part to the reluctance of big companies to invest in innovations for the long term. This scenario represents an excellent opportunity to create small‐ and medium‐sized enterprises that are highly specialized. A new wave of such ‘marine biotech enterprises’ based on modern bioprospecting would provide employment for highly qualified staff and the chance to pursue innovative, ‘high‐tech’ ideas at the cutting edge of modern science.

The complexity of the human gut microbiome has limited the development of microbiome‐based therapeutics (Van der Lelie *et al*., 2017). However, this could be an excellent opportunity to improve current technologies to further understand the term ‘microbiome complexity’ and create a new network of innovative enterprises and further job opportunities. Metagenome approaches, including next‐generation gene sequencing, bioinformatics, functional genomics, transcriptomics, metabolomics and other OMICS technologies, including the efficient engineering of bacteria chassis for heterologous expression as well as knowledge of cell‐to‐cell communication are all transversal disciplines that can be used outside the realm of the human body to interpret the role of other global microbiomes, including the marine microbiome.

The bacterial habitants of the human gut may control the health of our immune system, but the marine microbiome manages the health of life on earth, including the biogeological balances mitigating climate changes.

But what really is the marine microbiome?

The concept of microbial communities in the marine environment has gained increasing importance over the last few decades due to the abundance of living forms, their diversity and the critical role of the marine microbiome in determining the structure and function of oceans, and as a key player in the regulation of the carbon and nitrogen cycles and the balance of the global marine biomass. Some highlights from the Census of Marine Life (2010) (http://www.coml.org/) has revealed the astonishing fact that the number of bacteria in the open ocean exceeds 10^29^, representing more than 90% of the total marine biomass, with more than one million living bacteria in each millilitre of sea water. A single litre of seawater has been estimated to contain approximately 20 000 species with archaeal cell numbers rivalling those of bacteria as well as an astronomic number of eukaryotic cells and marine viruses and phages. In reality, nobody actually knows how many bacteria, archaea or eukaryotic microorganisms and viruses are currently living or would be able to survive in the ocean's dark depths. This question may remain unanswered for many decades.

This fascinating universe of different life forms is far from being a collection of static elements, but is continuously interacting amongst themselves and with their environment, developing strategies for survival beyond the wildest imagination of our rational human minds. Evolution has developed a myriad of enzymatic reactions and highly selective chemical weapons in response to *quorum sensing* signals. This adds an extra level of complexity to the understanding of the marine microbiome.

An example of successful ‘blue business’ that provides ‘proof‐of‐concept’ for exploiting marine chemical diversity in human health is demonstrated by the approval of the marine‐derived medicine Yondelis^®^ (trabectedin) for the treatment of certain types of soft tissue sarcomas and ovarian cancers. This compound has been developed by PharmaMar, a biopharmaceutical company focused on oncology and committed to research and development and taking inspiration from the sea to discover molecules with antitumor activity. Trabectedin belongs to the ecteinascidin family of antitumor compounds and was isolated from the tunicate *Ecteinascidia turbinata*. Other interesting marine‐derived compounds under development by PharmaMar for treating other cancers provide further evidence that the sea can be an extraordinarily rich source of new medicines with novel mechanisms of actions.

Early studies of the marine environment focused on natural products from invertebrates and tunicates and led to the isolation of several classes of bioactive natural products that were mainly small molecules such as polyketides and non‐ribosomal peptides. However, there is an increasing foundation for the rational suspicion, based on marine sponge/tunicate metagenomics, that such compounds although originally isolated from metazoan organisms are in actual fact of bacterial origin.

There are now many cases of molecules such as trabectedin, didemnins, onnamides, bryostatin, dolastatin, swinholides, calyculin A and other fascinating marine compounds where the putative gene clusters have been identified in symbionts or free‐living bacteria, thereby confirming the role of the marine microbiome as a producer of bioactive metabolites with potential applications in oncology. The paper of Wilson *et al*. (2014) is highly recommended and describes how they have identified a bacterial symbiont, living in sponges and harbouring a large number of gene clusters for secondary metabolites. The type of chemical scaffold produced is not restricted to anticancer bioactives, and other polyketide and peptide small molecules are continuously being discovered as anti‐infective or modulation agents for metabolic diseases.

Currently, genomic mining for polyketide synthases (PKS) and non‐ribosomal peptide synthetases (NRPS) improves the probability of success in drug discovery using marine microorganisms, both in isolated genomes and metagenomes. Over recent years, several computational tools have been developed for the analysis and prediction of specific classes of secondary metabolites. AntiSMASH (Blin *et al*., 2017) has served as a comprehensive web server and a stand‐alone tool for the automatic genomic identification and analysis of biosynthetic gene clusters of any type, with other more specific tools such as ClustScan, NP.searcher or SBSPKS focusing on non‐ribosomal peptide and polyketide biosynthesis pathways. Such bioinformatic tools are creating great expectations for future drug discovery by revealing the multitude of silent (cryptic) biosynthetic gene clusters.

In parallel, ever‐increasing research efforts are being directed towards personal care products based on marine microbial components. Cosmeceuticals, the combination of cosmetics and pharmaceuticals, is a booming business and revenues for this sector are expected to show two‐digit growth over the next few decades. The review by Martins *et al*. (2014) describes several examples of successful cosmeceuticals including the marketed product Abyssine^®^, composed mainly of a microbial exopolysaccharide (EPS), named deepsane and produced by a deep‐sea bacteria *Alteromonas*, isolated from a polychaete annelid living in a hydrothermal vent at a depth of 2600 m (Cambon‐Bonavita *et al*., 2002). This particular EPS effectively protects keratinocytes from inflammatory agents and is marketed for soothing and reducing irritation of sensitive skin against chemical, mechanical and ultraviolet B (UVB) aggression.

A further interesting example is provided by the pseudopterosins, tricyclic diterpene glycosides originally isolated from the Caribbean Sea whip *Pseudopterogorgia elisabethae*, and later reported to be metabolites of the dinoflagellate symbiont *Sympoidinium* sp. localized within the tissues of the sea whip (Mydlarz and Jacobs, 2004). The pseudopterosins are the main components of the cosmetic care product Resilience^®^ which possesses anti‐inflammatory and analgesic activities with the ability to inhibit PLA2 and degranulation and leukotriene formation in human neutrophils.

LIPOTEC (Barcelona, Spain) has launched SeaCode^®^, a mixture of extracellular glycoproteins (GPs) and other glucidic exopolymers produced by biotechnological fermentation of a *Pseudoalteromonas* sp. isolated from the intertidal coast of Antarctic waters. These GPs play a key role in cellular protein maintenance, cell‐to‐cell communication, stress recovery and as constituents of cell walls and are much sought after for skin care and reconstitution and other dermal and epidermal purposes.

Other exciting applications of the marine microbiome are provided by Corinaldesi *et al*. (2017) who describe several new examples of marine bacteria and fungi as producers of photo‐protective, anti‐ageing agents, bioemulsifiers and biosurfactants.

## Challenges and outlook

With the advent of molecular tools, the concept of marine biodiversity has been dramatically extended to cover all marine genome resources, with the number of microscopic life forms in the open ocean now thought to exceed 10^29^ and to represent more than 90% of the total marine biomass.

This incredible number of living organisms forms complex, equilibrated and dynamic ecosystems that use chemical signals to be in constant communication for natural and evolutionary adaptation. Some of these microbial products are already being marketed as pharmaceuticals, cosmetics and other high added‐value applications.

The discovery of bioactive marine natural products at the end of the last century, increased interest in marine bioprospection which has today evolved towards a more biotechnological prospection with the marine microbiome (free‐living microorganisms or those intimately associated to metazoans as symbionts) becoming an exciting source of raw materials for the discovery of high added‐value products.

The marine microbiome is an entire universe linking through constant signalling and communication. Chemical messages may induce the activation of unknown gene responses for defence or attack or modulate the microenvironment by sequestration of vital elements and ions or formation of biofilms to favour survival.

New research strategies to mimic natural conditions through the use of elicitors, co‐cultures, *quorum sensing* signalling and the specific modulation of the genomic regulation systems are innovative applications of modern synthetic biology that will provide a new and more rational approach for drug discovery.

The marine microbiome has enormous potential as the basis for new science in academia and new business opportunities. The discovery of new compounds with new applications will be the starting point for further new enterprises.

The ability to realize these opportunities and unlock the power of the marine microbiome requires new technologies and new tools. Deep‐sea sampling, metagenomics, next‐generation sequencing, genome mining and bioinformatics, innovative chassis for heterologous expression of large gene clusters, culture of fastidious organisms, induction of silent biosynthetic gene clusters, new analytical technologies, development of new screening platforms for unknown mechanism of actions and a large list of transversal biotechnologies provide just a few examples of the many developing technologies that will unlock the potential of the marine microbiome and play a crucial role in future marine science. Standardization of the methodologies used to generate and analyse the MEGA‐data of sequences of nucleic acids and proteins produced by metagenomics will also be important.

Future marine science will transform data into knowledge and the marine microbiome into new products. I hope we don't have to wait too long for this to happen.

## Conflict of interest

None declared.
